# Performance of the pooled cohort equation in South Asians: insights from a large integrated healthcare delivery system

**DOI:** 10.1186/s12872-022-02993-z

**Published:** 2022-12-23

**Authors:** Neha M. Mantri, Maqdooda Merchant, Jamal S. Rana, Alan S. Go, Seema K. Pursnani

**Affiliations:** 1 Department of Cardiology, Palo Alto Veterans Health Care System, Palo Alto, CA USA; 2 Division of Research, Kaiser Permanente, Oakland, CA USA; 3 Department of Health Systems Science, Kaiser Permanente Bernard J. Tyson School of Medicine, Pasadena, CA USA; 4 Department of Epidemiology, Biostatistics and Medicine, University of California, San Francisco, San Francisco, CA USA; 5 Department of Medicine, Stanford University, Palo Alto, CA USA; 6 Department of Cardiology, Kaiser Permanente Santa Clara Medical Center, 710 Lawrence Expressway, Dept 348, Santa Clara, CA 95051 USA

**Keywords:** ASCVD risk prediction, South Asian, Statin

## Abstract

South Asian ethnicity is associated with increased atherosclerotic cardiovascular disease (ASCVD) risk and has been identified as a “risk enhancer” in the 2018 American College of Cardiology/American Heart Association Guidelines. Risk estimation and statin eligibility in South Asians is not well understood; we studied the accuracy of 10-years ASCVD risk prediction by the pooled cohort equation (PCE), based on statin use, in a South Asian cohort. This is a retrospective cohort study of Kaiser Permanente Northern California South Asian members without existing ASCVD, age range 30–70, and 10-years follow up. ASCVD events were defined as myocardial infarction, ischemic stroke, and cardiovascular death. The cohort was stratified by statin use during the study period: never; at baseline and during follow-up; and only during follow-up. Predicted probability of ASCVD, using the PCE was calculated and compared to observed ASCVD events for low < 5.0%, borderline 5.0 to < 7.5%, intermediate 7.5 to < 20.0%, and high ≥ 20.0% risk groups. A total of 1835 South Asian members were included: 773 never on statin, 374 on statins at baseline and follow-up, and 688 on statins during follow-up only. ASCVD risk was underestimated by the PCE in low-risk groups: entire cohort: 1.8 versus 4.9%, *p* < 0.0001; on statin at baseline and follow-up: 2.58 versus 8.43%, *p* < 0.0001; on statin during follow-up only: 2.18 versus 7.77%, *p* < 0.0001; and never on statin: 1.37 versus 2.09%, *p* = 0.12. In this South Asian cohort, the PCE underestimated risk in South Asians, regardless of statin use, in the low risk ASCVD risk category.

## Background

South Asians (originating from India, Pakistan, Bangladesh, Nepal, Sri Lanka, Bhutan and the Maldives) make up one quarter of the world’s population and disproportionately account for more than half of the cardiovascular disease burden worldwide [1]. South Asians are at a heightened risk for premature atherosclerosis, with increased hospitalizations and deaths due to atherosclerotic cardiovascular disease (ASCVD) [2]. In the United States, data from the National Center for Health and Statistics show the highest proportionate mortality ratios in Asian Indian men (1.43) and women (1.12), compared with non-Hispanic Whites (NHWs) [3]. Traditional clinical ASCVD risk factors incompletely explain the heightened risk; in our Kaiser Permanente Northern California (KPNC) population, even after adjustment for traditional risk factors, compared with White adults, South Asian adults had an adjusted twofold higher odds of coronary heart disease (CHD) events [4].

I,n recognition of the increased ASCVD risk in South Asians, the 2018 Multi-Society Guideline on the Management of Blood Cholesterol and the 2019 American College of Cardiology/American Heart Association (ACC/AHA) Primary Prevention of Cardiovascular Disease Guideline define South Asian ethnicity as a risk-enhancing clinical factor to further improve primary prevention efforts [5–7]. A disproportionate burden of concomitant “risk enhancers,” such as a family history of premature CHD, metabolic syndrome, and elevated lipoprotein(a) levels, have previously been described in South Asians [8]. Quantifying the effect of these risk enhancers, in isolation or in combination, is not well understood. Aside from the QRISK risk assessment tools derived from and for the United Kingdom population, ASCVD risk calculators derived from and for South Asian populations are lacking [9]. Current North American population-specific ASCVD risk assessment tools are largely derived from White and Black volunteer cohorts in the United States [10–13]. In the limited studies where Asians have been included, the Asian population has not been disaggregated into Asian subgroups [14]. Furthermore, data on statin efficacy in South Asians is limited. The Mediators of Atherosclerosis in South Asians Living in America (MASALA) study is an ongoing prospective study that aims to provide further insight into cardiovascular risk factors in this high-risk ethnic group [15].

ASCVD risk estimation ultimately guides clinical decision-making regarding statin use and dose for primary prevention. Use of the Pooled Cohort Equation (PCE) has significantly expanded the number of people eligible for statin initiation [16]. Risk estimation in high risk groups who may already be on statins is not well understood, but becomes increasingly important given the high prevalence of statin use. Furthermore, risk estimation in higher risk groups, such as South Asians, may also provide indirect evidence of statin efficacy and insights into residual risk reduction strategies.

Risk estimation in South Asians using the pooled cohort equation (PCE) has shown variable results, with an overestimation of ASCVD risk in a composite group of Asians in our KPNC population (observed 17.2%, predicted 18.1%), and overestimation in Asian Indians in another Northern California healthcare system (observed 0.90%, predicted 1.2%) [17]. Similarly, in a population in Pakistan, risk estimation with the PCE overestimated risk as compared to use of a risk calculator incorporating coronary artery calcium score and high sensitivity C-reactive protein levels [18]. In the MASALA cohort, when using outcomes of subclinical atherosclerosis, the PCE demonstrated overestimation in the low and intermediate risk group, but stronger correlation in the high-risk group [19, 20]. In contrast, application of the PCE to the United Kingdom (UK) biobank cohort revealed underestimation of risk in those of South Asian, as compared to European, ancestry. Despite an increased ASCVD risk in South Asians (6.8% vs 4.4%, aOR 2.3, 95% CI 1.86–2.22) at a median of 11 years of follow-up, the calculated 10-years risk by the PCE was underestimated at 4.8% versus 6.0% in those of South Asian, versus European, ancestry, respectively [21].

In our current study, we aim to evaluate the accuracy of 10-years ASCVD risk prediction by the PCE, as well as statin eligibility and use, in South Asian adults receiving care within a large integrated health care delivery system. Given the large number of existing statin users in our KPNC population, in large part due to application of the PCE, and in an attempt to understand residual risk in this high risk ethnic group, we further stratify our sample by statin use.

## Methods

### Study sample

The source population was from KPNC, an integrated health care delivery system currently providing comprehensive care for > 4.5 million members throughout Northern and Central California. The KPNC membership is highly representative of the local surrounding and statewide population with regards to age, gender, race/ethnicity and socioeconomic status [22].

We conducted a retrospective cohort study of KPNC members identified as South Asian, based on ethnic origin from India (including Fijian Indians), Pakistan, Bangladesh, Nepal, Sri Lanka, Bhutan, and the Maldives. Granular self-reported race/ethnicity data were obtained from the KPNC Division of Research (DOR) Virtual Data Warehouse demographics database and from the Epic HealthConnect electronic medical record system; both systems include a list of all possible nationality options for entry (allowing for disaggregation of Asian subgroups). Additional inclusion criteria required having a complete lipid panel in 2006, no known clinical ASCVD before study entry in 2006, age 30–70 years, and complete 10-years follow-up (except if due to death). Exclusion criteria included end-stage renal disease, end-stage liver disease, lack of a blood pressure reading or lack of available smoking data at study entry.

### Outcome

Our primary outcome was a composite ASCVD outcome, including hospitalized myocardial infarction, hospitalized ischemic stroke, and cardiovascular death identified through December 31, 2016. Myocardial infarction and stroke were ascertained from comprehensive electronic health records using previously validated primary discharge diagnosis *International Classification of Diseases, Ninth* (ICD-9) or *Tenth* (ICD-10) Edition. Deaths were ascertained from health system electronic health records (including member proxy reporting), Social Security vital status files, and state death certificate data.

### Covariates

Covariates collected included: age at time of study entry, self-reported gender, fasting glucose, outpatient body mass index (BMI), total cholesterol, low density lipoprotein (LDL), high density lipoprotein (HDL), triglycerides, smoking status, diagnosed diabetes mellitus, diagnosed hypertension and receipt of lipid-lowering medications in the year prior to cohort entry. If multiple measurements were available, the most recently available laboratory data were used.

### Statistical analyses

Patients were classified into four 10-years predicted risk categories based on the ACC/AHA PCE risk calculator: low (< 5.0%), borderline (5.0 to < 7.5%), intermediate (7.5 to < 20.0%), and high ( ≥ 20.0%). In addition, the cohort was stratified based on prior statin use: never received statin therapy, initiated statin therapy during follow-up only, and received statin therapy at entry and during follow-up. We calculated the 10-years incidence of observed ASCVD events for each group and used binomial testing to compare this to the calculated predicted probability according to the PCE. All analyses were conducted using SAS statistical software, version 9.4 (Cary, N.C.) The KPNC Institutional Review Board approved this study, and a waiver of informed consent was obtained due to the nature of the study.

## Results

A total of 1852 eligible South Asian members were identified. Among patients receiving statin therapy at entry, 17 discontinued statin therapy due to side effects and/or individual preference and were excluded from further analysis. Our final analytic cohort included 1835 eligible South Asian adults. Among eligible adults, 773 (41.7%) members never received statin therapy at entry or during follow-up, 668 (36.1%) initiated statin therapy during follow-up only, and 374 (20.2%) received statin therapy both at entry and during follow-up. The average age of members was 49.2 ± 10.5 years and 50.9% being women. Nearly two-thirds of all patients were overweight and obese (Table 1).

**Table 1:** Baseline characteristics of south asian adults, stratified by statin exposure

Characteristic	Total (n = 1835)	Statin exposure status		
		Never user (n = 773)	Initiated during follow-up only (n = 688)	Received before and during follow-up (n = 374)
Age, mean (SD), year*	49.2 (10.5)	45.4 (10.5)	50.3 (9.7)	55.1 (8.7)
Self-reported gender*				
Women	934 (50.9%)	447 (57.8%)	321 (46.7%)	166 (44.4%)
Men	901 (49.1%)	326 (42.2%)	367 (53.3%)	208 (55.6%)
Body mass index, kg/m^2^*				
Normal (< 25)	609 (34.3%)	316 (41.9%)	197 (29.9%)	96 (26.7%)
Overweight (25–30)	790 (44.6%)	305 (40.5%)	307 (46.6%)	178 (49.4%)
Obese (≥ 30)	374 (21.1%)	133 (17.6%)	155 (23.5%)	86 (23.9%)
Smoking status**				
Current	180 (9.8%)	60 (7.8%)	72 (10.5%)	48 (12.8%)
Former	92 (5.0%)	28 (3.6%)	42 (6.1%)	22 (5.9%)
Never	1563 (85.2%)	685 (88.6%)	574 (83.4%)	304 (81.3%)
Diabetes mellitus*	417 (22.7%)	25 (3.2%)	184 (26.7%)	208 (55.6%)
Hypertension**	406 (22.1%)	140 (18.1%)	178 (25.9%)	88 (23.5%)
Other lipid-lowering medication use**	42 (2.3%)	6 (0.8%)	22 (3.2%)	14 (3.7%)
Lipoprotein level, mg/dl				
LDL*	118.7 (33.7)	113.4 (26.8)	133.8 (36.7)	102.0 (29.6)
HDL*	46.7 (11.8)	47.6 (12.6)	45.9 (11.3)	46.5 (10.8)
Triglycerides*	155.6 (91.3)	133.3 (78.4)	176.0 (97.4)	163.8 (94.7)
Total cholesterol*	196.3 (39.4)	187.5 (31.0)	214.7 (42.2)	180.7 (36.6)
Systolic blood pressure, mmHg*	125.1 (15.4)	122.3 (15.0)	126.8 (15.8)	127.8 (14.6)

*p < 0.0001, **p < 0.05

Members never receiving statin therapy and those initiating statin therapy during follow-up only were younger, compared to those who received statin therapy at entry and during follow-up. There was also a higher proportion of women in those never receiving statin therapy compared to the other exposure groups. The mean LDL for the total cohort was 118.7 ± 33.7 mmol/L, with the highest LDL in members on statin therapy only during follow-up (133.8 ± 36.6 mmol/L) and the lowest LDL in members on statin before and during follow-up (102.0 ± 29.6 mmol/L). Nearly one-fourth were diabetic, with the largest proportion in those receiving statins at entry and during follow up and one-fourth were on medication for hypertension, with the highest proportion in those initiating statin therapy during follow-up only. (Table 1).

At study entry, the distribution of ACC/AHA PCE 10-years predicted ASCVD risk among South Asian adults was as follows: 64.6% low risk (< 5%), 10.4% borderline risk (5.0 to < 7.5%), 20.0% intermediate risk (7.5 to < 20.0%), and 0.05% high risk (≥ 20.0%). In comparing predicted versus observed 10-years risks of ASCVD events among all patients, the ACC/AHA PCE significantly underestimated actual ASCVD risk in those classified as low or borderline risk (Table 2). In analyses stratified by statin exposure status, the ACC/AHA PCE underestimated risk across all risk categories and across all statin subgroups, with statistically significant differences observed in those considered low risk and either initiating statin therapy during follow-up only or receiving statin therapy at entry and during follow-up (Table 3).

**Table 2:** Comparison of 10-years observed vs. ACC/AHA PCE-predicted risks of ASCVD events in South Asian adults

AHA/ACC PCE 10-years predicted risk category	Composite outcome of myocardial infarction, stroke and presumed cardiovascular death						
	N	Predicted events (N)	Observed events (N)	Mean predicted risk (%)	Observed risk (%)	Relative risk (predicted: observed)	*p*
Low (< 5.0%)	1186	21.4	58	1.8	4.9	0.37	< 0.001
Borderline (5.0 to < 7.5%)	191	11.8	19	6.2	10.0	0.62	0.03
Intermediate (7.5 to < 20.0%)	368	44.1	52	12.0	14.1	0.85	0.21
High (≥ 20.0%)	90	24.4	30	27.1	33.3	0.81	0.18

**Table 3:** Comparison of 10-years observed versus ACC/AHA PCE-predicted risks of ASCVD events in South Asian adults, stratified by statin exposure status

AHA/ACC PCE 10-years predicted risk category	Composite outcome of myocardial infarction, stroke and presumed cardiovascular death						
	N	Predicted events (N)	Observed events (N)	Mean predicted risk (%)	Observed risk (%)	Relative risk (predicted: observed)	*p*
*Never user of statins (n = 773)*							
Low (< 5.0%)	621	8.5	13	1.4	2.1	0.67	0.12
Borderline (5.0 to < 7.5%)	53	3.2	5	6.1	9.4	0.65	0.32
Intermediate (7.5 to < 20.0%)	89	10.6	14	12.0	15.7	0.76	0.27
High (≥ 20.0%)	10	2.6	4	25.5	40.0	0.64	0.29
*Initiated statin therapy during follow-up only (n = 688)*							
Low (< 5.0%)	399	8.7	31	2.2	7.8	0.28	< 0.001
Borderline (5.0 to < 7.5%)	92	5.7	8	6.2	8.7	0.71	0.32
Intermediate (7.5 to < 20.0%)	161	19.2	20	11.9	12.4	0.96	0.84
High (≥ 20.0%)	36	9.7	12	26.9	33.3	0.81	0.38
*Received statin therapy at entry and during follow-up (n = 374)*							
Low (< 5.0%)	166	4.3	14	2.6	8.4	0.31	< 0.001
Borderline (5.0 to < 7.5%)	46	2.9	6	6.2	13.0	0.48	0.05
Intermediate (7.5 to < 20.0%)	118	14.3	18	12.1	15.3	0.79	0.30
High (≥ 20.0%)	44	12.2	14	27.6	31.8	0.87	0.53

## Discussion

In a large, contemporary, “real world” cohort, we demonstrated that the ACC/AHA PCE underestimated actual 10-years ASCVD risk in South Asians regardless of statin use, with the greatest difference and statistical significance observed in the low-risk ASCVD category. While our findings did not show statistical significance in the intermediate risk category, South Asian ethnicity has been identified in clinical practice guidelines as an ASCVD “risk enhancer” in evaluation of borderline and intermediate risk categories. Our findings suggest that South Asian ethnicity should be considered additionally in up-classifying risk in those currently classified as low-risk (< 5% 10-years ASCVD risk). Although not derived from data including South Asians, the use of the PCE risk calculator is recommended as part of contemporary clinical practice guidelines. Even though the PCE is not specifically validated in those on statin therapy, there is clinical utility in serial PCE assessment to guide ASCVD risk in patients with treated LDL, and, in fact, statin use is included in the commonly utilized ACC web and app-based “ASCVD Risk Estimator Plus” calculator. Furthermore, novel lipid lowering trials point to LDL reduction, regardless of statin use, as predictive of ASCVD risk. Lower LDL-C levels, with statin and/or non-statin therapies, are associated with lower rates of major coronary events [23]. Given our expanding population of statin users, accurate risk estimation and modification are even more essential as we expand the clinical armamentarium of non-statin therapies. We therefore also sought to pragmatically evaluate risk prediction, stratified by statin use, in our South Asian cohort. In those members who were never on statins during the study period, the risk was underestimated across all groups. In the intermediate and high-risk groups, where statin prescribing would be recommended, the risk was underestimated, in addition to the low and borderline risk categories, where statin prescribing may be variable based on current recommendations. In those members on statin therapy at entry and during the follow up period, risk was again underestimated across groups, particularly in the low and borderline risk groups; the risk underestimation could be related to a greater burden of concomitant risk factors (age, current smokers, overweight BMI, and diabetics) in this group. Finally, in those members who were started on statins during the follow up period, risk prediction was again underestimated across categories, but appeared to be best calibrated in the intermediate risk category. Risk underestimation across members on statin therapy necessitates a better understanding of residual ASCVD risk in South Asians.

South Asians experience a known higher burden of premature CHD compared with NHW and other selected racial/ethnic groups. We have previously reported a disproportionate burden of CHD among South Asian adults within KPNC, which is not fully explained by measurable clinical risk factors, such as diabetes, hypertension, dyslipidemia, and obesity [4]. In the UK Biobank cohort, hypertension, diabetes, central adiposity, and lifestyle factors only partially explained the disparate risk seen in those participants of South Asian versus European ancestry [21]. Additional potential explanatory risk factors include those factors that are difficult to assess using typical epidemiologic methods, including weight distribution, dietary patterns, family history and genetic factors, and social determinants of health. Identifying the subgroup of South Asian adults who are at high enough ASCVD risk to warrant primary prevention therapies remains challenging.

Commonly used cardiovascular risk prediction models built to estimate ASCVD risk in the North American population include the Framingham Adult Treatment Panel (ATP) III model, the Multi-Ethnic Study of Atherosclerosis (MESA) model, and the ACC/AHA Pooled Cohort Equation (PCE). External validation has shown inconsistent calibration [24–27]. The Framingham Heart Study, initiated in 1948 in Framingham, Massachusetts, consisted of a cohort of predominantly middle class, White American adults from urban settings. The Framingham Risk Score (FRS) prediction model was developed in 1976 to estimate 10-years risk of cardiovascular disease using a simple point-based system. While the FRS was validated in 5-years cardiovascular risk prediction in NHW and African American (AA) men and women, it performed poorly in risk prediction in Japanese American men, Hispanic men, and Native American women, with an overestimation of risk in these groups [28, 29]. The MESA study was a community-based, prospective cohort study of 6,814 men and women aged 45–84 years from six United States communities of varied socioeconomic and racial backgrounds. Although the MESA study included Chinese Americans, Black, Non-Hispanic Whites, and Hispanic adults, South Asians were not included. Similarly, the 2013 ACC/AHA PCE was validated for only NHW and Black adults from volunteer cohorts, with a recommendation to use the NHW group to classify risk in all other non-Black racial/ethnic groups [5]. The ongoing MASALA study, a population of exclusively South Asians living in the United States, will hopefully provide additional insights in risk estimation of clinical ASCVD events in this high risk population.

The strengths of our study include use of a “real-world” population that is more likely to represent typical patients in U.S. communities who may be considered for preventative therapies; robust 10-years follow-up for all included members; and the assessment of risk in patients with and without statin therapy, which is a contemporary dilemma considering an expanding pool of statin users.

Limitations of our study include the inability to serially assess risk factor prevalence and severity, as well as statin prescribing practices during the 10-years follow up; generalizability of findings to South Asian populations in other geographic regions; and treatment selection bias in the statin groups. Exploration of birth origin, migration patterns, lifestyle factors, social determinants of health, and measures of subclinical atherosclerosis, such as coronary artery calcium, were not available. Variation in individual prescriber practices and adherence to statins, which have been previously shown to vary by race/ethnicity, were not explored [30–32]. In addition, real-world quality of life outcomes, which are closely related to patient adherence, were not available [33–35].

There are currently no validated South Asian- specific calculators or quantitative recommendations for ASCVD prediction and guidance regarding statin therapy or other primary prevention risk reduction strategies. We found the PCE to significantly underestimate 10-years ASCVD risk in our South Asian cohort, particularly in those currently classified as low risk. While current guidelines do not recommend statin therapy or assessment of risk enhancers in the “low risk” ASCVD group, our findings suggest that individualized risk assessment, with incorporation of South Asian ethnicity and other prevalent concomitant risk enhancers in this population, may help identify individuals that may benefit from intensification of lifestyle changes and initiation of statin therapy to reduce ASCVD risk. Furthermore, additional studies are required to assess residual cardiovascular risk and treatment in this high risk ethnic group.

## Conclusion

In this large, community-based South Asian cohort, the ACC/AHA PCE underestimated ASCVD risk, regardless of statin use, particularly among the subgroup of patients currently classified as low ASCVD risk. Further studies are required to improve risk prediction and understand residual risk in this high-risk ethnic group and to identify effective primary prevention strategies.

## Data Availability

The data that support the findings of this study are available from Kaiser Permanente but restrictions apply to the availability of these data, which were used under license for the current study, and so are not publicly available. Data are however available from the corresponding author (Dr. Pursnani) upon reasonable request and with permission of Kaiser Permanente.

## Abbreviations

ASCVD: Atherosclerotic cardiovascular disease
PCE: Pooled cohort equation
ACC/AHA: American College of Cardiology/American Heart Association
KPNC: Kaiser Permanente Northern California
